# Degree of conversion and residual monomer elution of 3D-printed, milled and self-cured resin-based composite materials for temporary dental crowns and bridges

**DOI:** 10.1007/s10856-023-06729-z

**Published:** 2023-05-12

**Authors:** Eva Berghaus, Thorsten Klocke, Reinhard Maletz, Svea Petersen

**Affiliations:** 1 Laboratory of Chemistry and Surface Modification, University of Applied Sciences Osnabrück, Osnabrück, Germany; 2 Department of Material Science and Medical Engineering, Faculty of Mechanical Engineering and Marine Technology, University of Rostock, Rostock, Germany

**Keywords:** HPLC, FTIR, CAD/CAM, Additive manufacturing

## Abstract

The aim of this work was to investigate the elution of residual monomers as a function of the manufacturing process, which are CAD/CAM manufacturing, self-curing and 3D printing. The experimental materials used consisted of the base monomers TEGDMA, Bis-GMA and Bis-EMA and 50 wt.% fillers. Additionally, a 3D printing resin without fillers was tested. The elution of the base monomers into the different media (water, ethanol and ethanol/water (75/25 vol. %)) at 37 °C over a period of up to 120 d as well as the degree of conversion (DC) by FTIR were investigated. No monomer elution could be detected in water. Most residual monomers in both other media were released from the self-curing material whereas the 3D printing composite released relatively little. The CAD/CAM blanks released hardly any quantitatively detectable amounts of monomers. Relative to the base composition, TEGDMA eluted less than Bis-GMA and Bis-EMA. DC did not correlate with residual monomer release; thus, leaching was determined not only by the amount of residual monomers present but by further factors as possibly network density and structure. The CAD/CAM blanks and the 3D printing composite showed similar high DC but lower residual monomer release from the CAD/CAM blank, likewise the self-curing composite and the 3D printing resin exhibited similar DC but different monomer elution. In terms of residual monomer elution and DC, the 3D printing composite seems promising as a new material class for the use as temporary dental crowns and bridges.

**Graphical Abstract:**

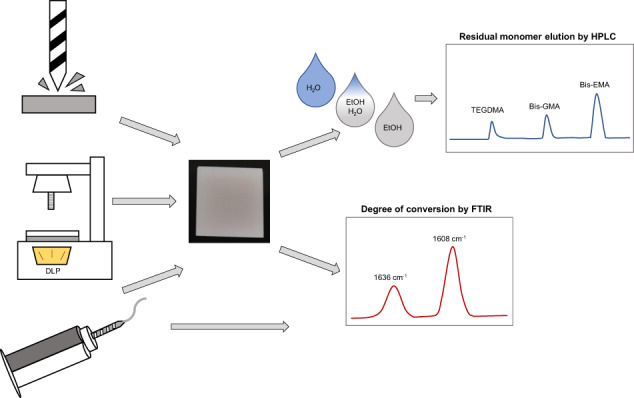

## Introduction

The use of methacrylate-based composites in dentistry began in the 1960s with their use as filling materials [1]. Since that time, there have been many developments in the monomer-filler composition of the materials resulting, among other things, in materials applicable for prosthetic restorations as crown and bridge materials. The use is not only limited to short-term applications, but also composites as permanent restorations become more and more common. For temporary use, the composites are often used as a self-curing polymer. For both temporary and permanent use, the materials are commonly produced as blanks and processed via subtractive CAD/CAM fabrication by milling. The materials for these two applications have been established on the market for many years and their properties are well researched [2–4]. In recent years, the spectrum of processing technologies has expanded to include additive manufacturing. With regard to the fabrication of polymer-based crowns and bridges, photoinduced manufacturing of liquid resins can be used. The stereolithography (SLA) or the digital light processing (DLP) are examples of these 3D printing technologies [5–7].

In the context of dental composites for use as crown and bridge materials, frequently methacrylate-based resins like triethyleneglycoldimethacrylate (TEGDMA), urethanedimethacrylate (UDMA), bisphenol A glycidyldimethacrylate (Bis-GMA) or 2,2,-bis(4-(2-Methacryl-oxyethoxy)phenyl)propane (Bis-EMA) are used. Ideally, these monomers should fully polymerize into a network during processing. In reality, however, not all monomers in the mixture are crosslinked into the network and are therefore present unbound. These remaining monomers and also initiators or other additives can either be sterically hindered within the network or eluate into a surrounding liquid medium. The elution of non-crosslinked components from dental resin-based materials into saliva and thus potentially into the system of the human body is a critical factor in the development and application of these materials. Residual monomers are suspected, for example, of causing allergies and hypersensitivities [8] in patients or of having cytotoxic, mutagenic and estrogenic effects on cells [8–12]. The extent of the elution depends, for instance, on the elution medium, mechanical stress, aging, hydrolysis or enzymatic degradation [13–16]. Regarding the elution media different solvent sets are used in literature [17–20]. Łagocka et al. [21] described a set of distilled water, a mixture of ethanol/water (75/25 vol. %) and pure ethanol, where water should simulate the intraoral environment, the mixture of ethanol/water is recommended by the US FDA to be more corresponding to oral cavity conditions and pure ethanol is used as a worst-case scenario to penetrate the matrix of composites. To estimate the total residual monomer content, the degree of conversion (DC) is measured as a further parameter along with monomer release [22, 23]. The rate and the degree of conversion reached depends on the size of the monomers, the number of functional units, the temperature and the initiator system. In the case of photoinduced polymerization, furthermore, on wavelength, light intensity and irradiation time [24, 25]. Although when the same monomer composition is applied for all manufacturing methods, a dependence of the conversion rate but also of the DC on the manufacturing method is assumed. Different initiator systems and processing conditions with associated varying flow properties of the monomers are probable reasons.

The elution of residual monomers from self-curing and light-curing composites [21, 26] and from composites for CAD/CAM processing [16, 19], as well as the correlation to their polymerization conversion [22, 23], has already been described in literature. For the relatively new technology of additive manufacturing of crown and bridge material from liquid resins, no studies concerning the elution of residual monomers have been reported. To the best of our knowledge, publications have so far been limited to polymer resins for splints [27] and surgical guides [28] or comparing other material properties [29]. In this context, the present study aims to characterize polymerization conversion and residual monomer elution in dependence of the different fabrication methods: conventional self-curing, milling and 3D printing. To accomplish this, experimental composites with the same monomer matrix, filler particles and monomer-filler ratio for all processing methods are used. Additionally, an unfilled 3D printing resin is used.

The null hypotheses tested were: (i) there is an elution of the matrix monomers from cured samples into the solvent; (ii) the eluted monomer concentration depends on the storage medium; (iii) the DC depends on the fabrication method used; (iv) the monomer release depends on the fabrication method (possibly in correlation to DC), and (v) the elution of monomers from the 3D printing materials and the DC varies with filler content.

## Materials and methods

### Materials

The experimental composites used were provided by VOCO and consisted of 50 wt.% filler (5 wt.% fumed silica, 95 wt.% dental glass) and 50 wt.% monomer matrix. The monomer matrix was composed of 12 wt.% TEGDMA, 35 wt. % Bis-GMA and 53 wt.% Bis-EMA. Depending on the processing method (3D printing or milling/conventional self-curing), different initiator systems and additives were added to the composites by the manufacturer. The material for milling and the one for conventional self-curing had the same composition. Additionally to these composites, an unfilled resin of the same monomer ratio was prepared for 3D printing (see Table 1).

**Table 1:** Overview of the materials used in this study, its reaction mechanisms and sample preparation according to the section materials and methods

Manufacturing	Filler content [%]		Type	Curing mechanism	Operator	Sample preparation of plates (14 × 14 × 2 mm) for measurement of residual monomer release and DC of cured plates	Sample preparation for DC measurement of uncured pastes
CAD/CAM	50	Composite		Redox	Industrial partner	Curing in strand form, cutting, milling, polishing	—
Self-curing	50	Composite		Redox (two cartridge system)	Prepared by authors	Mold casting 37 °C, removed from mold 24 h, 37 °C, milling, polishing	Paste as two cartridge system mixed directly before measurement, curing observed via FTIR over time
3D printing	50	Composite		Photoinitiation	Industrial partner	3D printed 385 nm, 50 µm layer thickness, washed in isopropanol, post-curing, milling, polishing	Light cured with LED curing unit (420–480 nm), curing observed via FTIR over time
	0	Resin		Photoinitiation	Industrial partner	3D printed 385 nm, 50 µm layer thickness, washed in isopropanol, post-curing, milling, polishing	Light cured with LED curing unit (420–480 nm), curing observed via FTIR over time

### Sample preparation

For the residual monomer release study and the measurement of the degree of conversion by FTIR square samples with the final dimensions of 14 × 14 mm ± 0.2 mm and a height of 1.9 mm ± 0.1 mm were prepared (see Table 1). The CAD/CAM blank was fabricated by VOCO and cut into plates with the ISOMET 4000 linear precision saw (Buehler) with a feed rate of 5 mm/min at 5000 U/min under water cooling. Sample plates (14 × 14 × 2 mm) were 3D printed with the SolFlex 350 DLP 3D printer (VOCO) with a wavelength of 385 nm and a layer thickness of 50 µm. During the process of 3D printing, heat develops in the material bath. This temperature is not controlled by the printer used and, from experience, is slightly above room temperature (30–40 °C). Afterwards the 3D printed samples were washed two times with isopropanol for two minutes per wash cycle. 3D printed samples were post-cured in two cycles with 2000 flashes with the light curing unit Otoflash G171 (VOCO), as industrial standard procedure. The self-curing composite was supplied as a two-component system in cartridges. Samples were generated by using a 14 × 14 × 2 mm stainless steel frame between two glass plates as mold. Isolation between the glass plates and the composite was obtained by a polymer foil. The self-curing polymer composite was injected into the mold by a dispenser (DS50, VOCO) and slight pressure was applied to the glass plates by using two foldback clips per mold to reduce pore formation. As polymerization started directly after mixing, the samples were placed in an incubator (Memmert) at 37 °C for five minutes with the molds and clips. After five minutes the molds were detached and the samples remained in the incubator for 24 h. All specimens, independent of previous preparation were shaped with sandpaper (360 and 600 grit), if necessary, and finely ground with 1000 grit sandpaper. The samples were polished with a 1 µm grain size polish and lubricant on a DAP 7 (Struers). After polishing, the samples were first cleaned with distilled water and cotton, then placed in an ultrasonic bath (Sonorex RK 100 H, Bandelin) with distilled water for three minutes and patted dry with a cellulose tissue. Size and weight of each sample were measured (Micrometer: MDC-25XS, Mitutoyo; Scale: MC1 Analytic AC 210 S, Sartorius).

## Methods

### Residual monomer release

To study the residual monomer release, five samples per material were used for each kind of storage media. Three different types of media were used: double distilled water, ethanol and a mixture of 75 vol. % ethanol with 25 vol. % double distilled water. One sample each was placed in a 10 mL vial with 2 mL storage media and stored at 37 °C under shaking (Hei-MIX Incubator 1000, Heidolph Instruments). All media was removed from the samples at the time points 1, 6, 24 h, 3, 6, 10 d, replaced with fresh media and analyzed by HPLC analysis. Additionally, long time release of monomers was measured in ethanol (worst-case scenario) at the time points 21, 37, 59, 92 and 120 d.

### HPLC analysis

Before analysis, all elution media were filtered with a 0.2 µm syringe filter. High pressure liquid chromatography (HPLC) analyses were performed as triplicates with an Agilent 1260 series HPLC, equipped with a Brownlee SPP C18, 2.7 µm, 3 × 100 mm column with additional pre coloum, with DAD detector at 205 nm wavelength. A method according to Łagocka et al. was used [21] and was defined as follows. As elution media acetonitril/double distilled water in the isocratic ratio 70/30 was used. Flow rate was set to 1 mL/min with a column temperature of 20 °C and an injection volume of 2 µL. For qualitative and quantitative evaluation of the elution, linear calibrations of the base monomers TEGDMA, Bis-GMA and Bis-EMA were prepared at each measurement day. TEGDMA and Bis-GMA each showed a substance peak at the retention times 0.6 min and 0.95 min, respectively. Due to its chemical structure, pure Bis-EMA generated several peaks in the chromatogram. The peak at 3.65 min was used for evaluation. Two Bis-EMA peaks retarded simultaneously with the Bis-GMA at 0.95 min. For quantitative evaluation of the eluates, the peak areas were subtracted from each other and evaluated by means of a resulting calibration curve. The confidence interval, the limit of determination and the limit of detection of the calibration curves were calculated and applied with a statistical certainty of 95% in each case. The resulting limit of quantification, depending on the day of measurement, was between 0.09 mg/L and 0.76 mg/L for TEGDMA, between 0.04 mg/L and 0.56 mg/L for Bis-GMA, and between 0.07 mg/L and 1.67 mg/L for Bis-EMA.

### Degree of conversion by FTIR

FTIR spectra of all cured samples and the uncured material of the self-curing composite and both 3D printing material (see Table 1) were measured with the NICOLET iS10 (Thermo Fisher Scientific) and evaluated with the OMNIC 9 (version 1.09, Thermo Fisher Scientific) software. Two spectra per sample of five cured samples (14 × 14 × 1.9 mm) and three spectra of the uncured pastes (a drop of ~0.5 mL) of each material were measured. The serial spectra measurements of the polymerization process of the uncured pastes over 15 min were recorded on a NICOLET 5700 (Thermo Fisher Scientific) and analyzed using OMNIC 9 (version 1.27, Thermo Fisher Scientific) software. This measurement was not possible for the CAD/CAM blanks as no uncured paste was available and is identical to the self-curing material. For both instruments the ATR attachment Smart Endurance (Thermo Fisher Scientific) was used. Polymerization of the 3D printing materials were initiated for 60 s by a high-performance LED curing unit Celalux 2 (Voco), which operates in the spectral range of 420–480 nm with a light intensity of 1000 mW/cm², during the series measurements. The wavelength used here to initialize polymerization thus differs from the wavelength of the 3D printer used to produce the samples. Nevertheless, with the help of this measurement, a comparison can be made between the filled composite and unfilled resins for additive manufacturing.

To calculate the degree of conversion (DC) [%], the procedure of Herrera-González et al. [30] was followed. Instead of the peak height, the peak area (PA) at 1608 cm^−1^ and 1636 cm^−1^ from the spectra of the cured (c) and uncured samples (uc) was used and set into relation for calculation (Equation 1).

Equation 1: Degree of conversion (DC) [%] calculated by the peak areas (PA) from cured and uncured samples at 1608 cm^−1^ and 1636 cm^−1^ after Herrera-González et al. [30]

$$DC = \left( {1 - \frac{{PA1636_c/PA1608_c}}{{PA1636_{uc}/PA1608_{uc}}}} \right) \ast 100{{{\mathrm{\% }}}}$$

### Statistics

Statistical analysis and artwork were conducted with the statistic software Prism (GraphPad). Significant differences between the mean values were determined using a one-way ANOVA with significance level ≤0.05 (Tukey test) for data from the degree of conversion measurement by FTIR for cured samples. Normal distribution was tested with the D´Agostino & Pearson test, variance homogeneity with the Brown-Forsythe test. For the comparison of the residual monomer release after 245 h from the different materials in the media ethanol and ethanol/water the Mann–Whitney test with significance level ≤0.05 was used.

## Results

### Residual monomer release

**Fig. 1:**
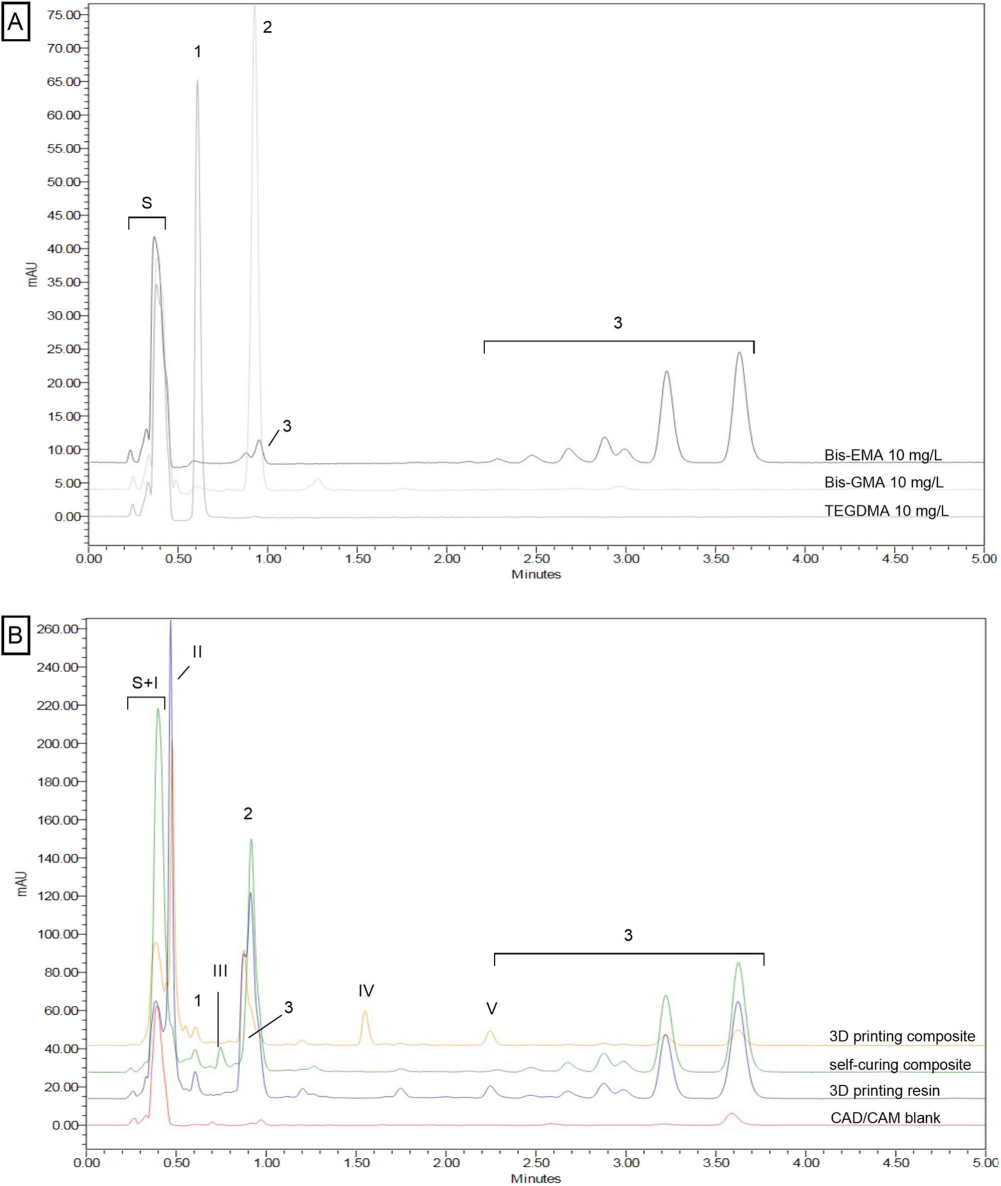
Chromatograms recorded by HPLC-DAD at 205 nm (**A**) of the pure monomers TEGDMA (Peak 1), Bis-GMA (Peak 2), and Bis-EMA (Peaks 3) with a solvent peak (S) at the time of injection. And (**B**) of eluates from the samples CAD/CAM blank (red), self-curing composite (green), 3D printing resin (blue) and 3D printing composite (yellow) stored in ethanol after 21 d. In addition to the peaks of the monomers (1–3), unknown substances labeled with (I-V) elute

The three monomers of interest could be separated and calibrated with the HPLC method used. The following retention times resulted: TEGDMA—0.6 min (1), Bis-GMA—0.95 min (2), Bis-EMA—3.65 min (3) (cf. Fig. 1A). Bis-EMA exhibited multiple elution peaks, two of them overlap with the peak of Bis-GMA. For calibration and evaluation of Bis-GMA, the corresponding area of the Bis-EMA peak was subtracted as described in the chapter “HPLC analysis”. Figure 1B shows the chromatograms of the eluates in ethanol after 21 d as an example. The peaks of the monomers could be found in the chromatograms of the eluates of the samples. In addition, further unknown substances eluted, whereby these differ between the production types of the samples. It is striking that the peak of the solvent (S) is very pronounced in the self-curing composite. It can be assumed here that another substance (I) eluted at the same time as the solvent. Another unknown substance (II) eluted from the two 3D printing materials shortly after the solvent (S), so that the peak areas overlap. These two phenomena influence the resolution of the TEGDMA peak (1). Other unknown substances eluted from the self-curing composite (III) and from the 3D printing composite (IV and V), whereby substance V was also found in the 3D printing resin.

The four different materials showed different kinetics for the release of residual monomers in dependence of the elution media and the type of monomer. The elution in water of TEGDMA, Bis-GMA and Bis-EMA for all tested materials at the selected time points was below the quantitative limit of detection. Likewise, the elution from the CAD/CAM blanks were mostly below limit of detection independent of the elution media and could only be quantified in ethanol or ethanol/water for very few points of time and samples.

**Fig. 2:**
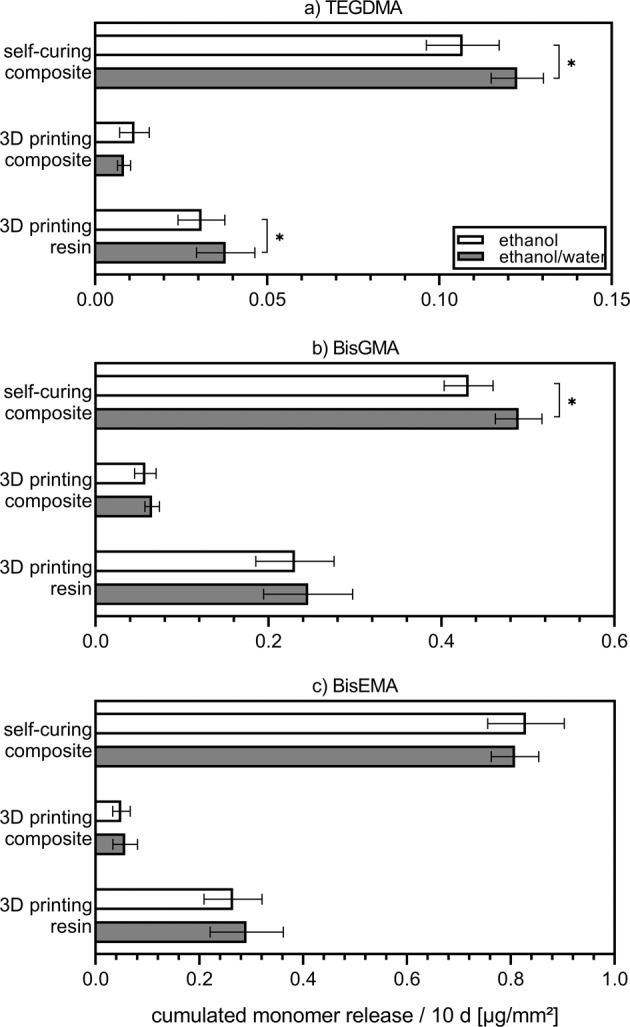
Cumulated release of the residual monomers TEGDMA (**a**), Bis-GMA (**b**) and Bis-EMA (**c**) over a period of 10 d normalized to the specimen’s surface area [µg/mm²]. The mean value with standard deviation of monomer release from two 3D printing materials and a self-curing composite in ethanol and ethanol/water as elution media are shown (*n* = 5). Significant differences (*p* ≤ 0.05) between the amount of elution in the two media are marked with a *

The cumulated release over 10 d of the three residual monomers measured, TEGDMA, Bis-GMA and Bis-EMA, in ethanol and ethanol/water is shown in Fig. 2. As the detected concentration of monomers is set as a function of specimen surface area [µg/mm²] the numbers can be compared directly between the materials tested and the elution media. As a slight trend, more elution of monomers in the ethanol/water mixture than in ethanol can be observed over almost all material/elution media combinations with exception of TEGDMA from the 3D printing composite and Bis-EMA from the self-curing composite. The higher release of monomers in ethanol/water mixture is significant (*p* ≤ 0.05) for TEGDMA from the self-curing composite and the 3D printing resin as well as for Bis-GMA from the self-curing composite. Regardless of the elution media, the release of residual monomers after 10 d was the highest from the self-curing composite followed by the release from the 3D printing resin. The samples from the 3D printing composite showed the least detectable monomer release whereas almost no quantitative determination could be made for the CAD/CAM blanks. For the milled samples only Bis-EMA was detectable as eluate in ethanol/water at the point of time of 10 d, so no cumulation was possible. Elution in ethanol could be measured for a few samples after 6 h, 3 d and 10 d but no descriptive cumulation could result from this data. As can be seen in Fig. 2 the most released monomer in terms of quantity in ethanol and ethanol/water was Bis-EMA, followed by Bis-GMA and TEGDMA. Complementary to Fig. 2, Table 2 lists the cumulated release of the residual monomers over a period of 10 d [µg] in correlation to the starting polymerizable amount of monomer [wt. ppm]. In order to consider the mere release from the monomer content, in this calculation the filler content was subtracted. It is also found that more monomer is released from the self-curing composite than from the 3D printed materials. In the latter, the unfilled resin releases more monomer than the filled composite.

**Table 2:** Cumulated mass release of the residual monomers TEGDMA, Bis-GMA and Bis-EMA over a period of 10 d [µg] in correlation to the starting polymerizable amount of monomer [wt. ppm]

monomer	self-curing composite [wt. ppm]		3D printing composite [wt. ppm]		3D printing resin [wt. ppm]	
	ethanol	ethanol/water	ethanol	ethanol/water	ethanol	ethanol/water
TEGDMA	1508	1732	222	163	292	355
Bis-GMA	2089	2371	389	436	742	789
Bis-EMA	2652	2585	219	252	563	617
Total	2318	2408	279	306	593	646

In the calculation, the filler content was subtracted in order to consider only the mere release from the monomer content

The percent release of the monomers after 10 d in ethanol and ethanol/water are shown in Table 3. Relative to the base composition, TEGDMA eluted less than Bis-GMA and Bis-EMA for all materials observed. Proportionally more Bis-EMA eluted from the self-curing composite while more Bis-GMA eluted from the two 3D printing materials.

**Table 3:** Cumulated release of the residual monomers TEGDMA, Bis-GMA and Bis-EMA over a period of 10 d [wt. %]

monomer	basic composition [wt. %]	self-curing composite [wt. %]		3D printing composite [wt. %]		3D printing resin [wt. %]	
		ethanol	ethanol/water	ethanol	ethanol/water	ethanol	ethanol/water
TEGDMA	12	7.8 ± 0.9	8.6 ± 0.5	9.6 ± 3.5	6.4 ± 1.4	5.9 ± 1.2	6.6 ± 1.4
Bis-GMA	35	31.5 ± 2.0	34.5 ± 1.8	48.4 ± 10.1	49.9 ± 6.1	43.8 ± 8.3	42.7 ± 8.6
Bis-EMA	53	60.6 ± 5.2	56.9 ± 3.1	41.9 ± 13.6	43.7 ± 17.4	50.3 ± 10.2	50.7 ± 11.9

The mean value with standard deviation of the proportionate monomer release from two 3D printing materials and a self-curing composite in ethanol and ethanol/water as elution media are shown (*n* = 5) in comparison to the basic monomer composition

**Fig. 3:**
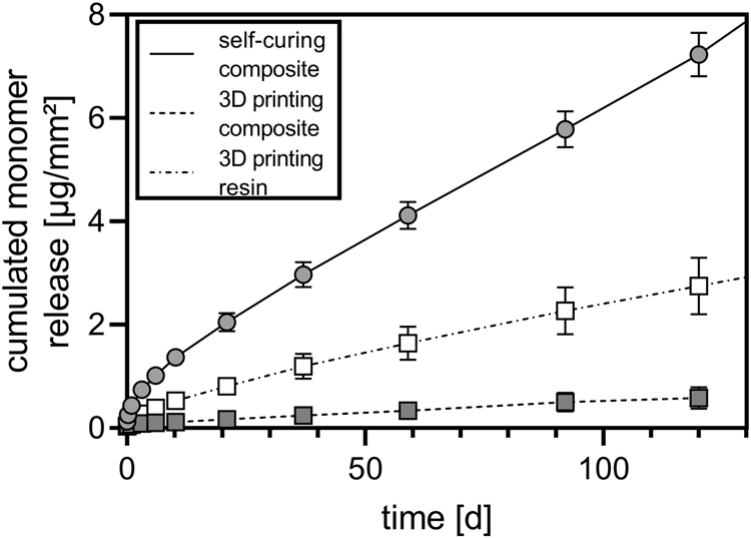
The amount of all released monomer per specimen’s surface area [µg/mm²] (mean value with standard deviation, *n* = 5) as a cumulation of the release of TEGDMA, Bis-GMA and Bis-EMA from two 3D printing materials and a self-curing composite in ethanol over a period of 120 d

The amount of all released monomer per specimen’s surface area [µg/mm²] (cumulation of TEGDMA, Bis-GMA and Bis-EMA) in ethanol over a period of 120 d is illustrated by Fig. 3. Over a longer period of time, the most monomer eluted from the self-curing composite, followed by the 3D printing resin and the 3D printing composite. The release from the CAD/CAM blanks again were under the limits of quantification for most points in time so no cumulative curve could be drawn. Monomer release from self-curing composite and the 3D printing resin in ethanol were additionally measured over a total period of nearly six months. The cumulated monomer release from the self-curing composite amounted 10.6 µg/mm² and from the 3D printing resin 3.7 µg/mm² (data not shown).

### Degree of conversion

**Fig. 4:**
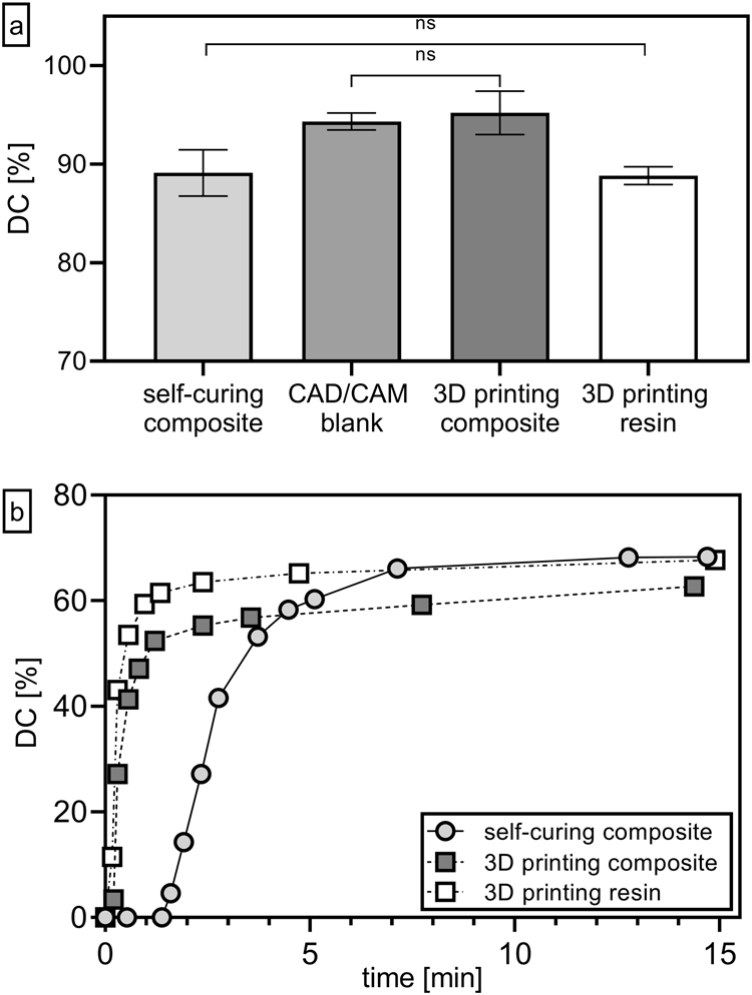
**a** Mean value and standard deviation (*n* = 5) of the Degree of conversion (DC) [%] of the cured samples (see “Sample preparation”). Non-significant differences (*p* > 0.05) between DC are marked with “ns”. **b** Degree of conversion (DC) [%] over time [min] during curing of the two 3D printing materials and the self-curing composite

The degrees of conversion (DC) of the sample plates (see “Sample preparation”) measured by FTIR are shown in Fig. 4a. The samples made by milling (CAD/CAM) and the 3D printed composite showed the highest DC with a mean value of 94.3%, respectively 95.2%, and are not significantly different from each other. The same material composition reached a significant lower DC of 89.1% in average with the processing as a self-curing material. A similar DC was measured for the unfilled 3D printing resin.

For the uncured paste of the self-curing composite and the two 3D printing materials the DC could be measured over time (Fig. 4b). Although the light source used (LED with 420–480 nm wave length) did not correspond to the one in a DLP printer (485 nm), the polymerization kinetics of both the 3D printing materials were comparable to each other and were faster than those of the self-curing composite. The self-curing material reached a DC over 60% after approximately five minutes whereas the unfilled 3D printing resin reached this percentage after one minute. For all three materials tested here the measured DC after 15 min do not reach the ones of the readily cured samples (Fig. 4a, b). Especially the 3D printing composite showed lower DC values. For the 3D printed materials, this can be attributed to the deviating light source of the initialization. Regarding the self-curing composite, the solid samples (Fig. 4a) were cured for 24 h at 37 °C, which is significantly higher than the 15 min at room temperature observed here.

## Discussion

The elution of the monomers in water can only be demonstrated qualitatively within the scope of this work. In the chromatograms, peaks can be assigned to TEGDMA and Bis-GMA but not for all materials and all points of time. Peaks of Bis-EMA are even less detectable. Quantification of the three monomers in water is not possible for any of the materials at the selected points of time. The low elution of Bis-GMA in water was already described by Łagocka et al. [21]. According to them a quantitatively detectable elution of TEGDMA in water could have been possible in the selected time intervals. Łagocka et al. detected an amount over 3 mg/L TEGDMA (sample size: 5 mm diameter, 4 mm height) eluting from flowable bulk-fill composite resins in 0.5 mL distilled water after 1 h storage time. Elution in ethanol and ethanol/water is much more prominent than in water, which was also already described [18, 21, 31], and similar to each other with a slight trend of more monomer elution in ethanol/water. Nevertheless, in literature the use of ethanol as elution medium is often used to detect the maximum amount of leachable monomers, whereas a mixture of ethanol/water is described to be more clinic relevant [21]. The release of residual monomers is detected for all materials and can be quantified for the self-curing composite and both 3D printing materials. The degree of conversion can be compared for all four materials, showing the highest value for the CAD/CAM blanks and the 3D printing composite. The rate of the degree of conversion can be observed for the 3D printing materials and the self-curing material.

The barely detectable elution of residual monomers in water (resp. salvia) could already be confirmed by several studies [32–34]. The low tendency of the monomers to dissolve in water can be explained by their hydrophobic structures, which are particularly pronounced in Bis-EMA and Bis-GMA [17]. The trend towards an increased influence on material aging by ethanol/water compared to ethanol, which would also increase monomer release, could also be observed by Wu et al. [33] based on the hardness reduction of the Bis-GMA matrix. The kinetics of residual monomer elution approximate those of other studies such as Łagocka et al. [21] or Nalçaci et al. [35]. Here, too, the highest elution by volume per time period was measured within the first hours. It is striking that in the studies presented here, with respect to the composition of the monomer matrix, TEGDMA is less involved in the elution than the other two monomers in all sample series (ethanol and ethanol/water after 10 d). This can possibly be explained by the smaller molecular size of the monomer, which leads to a higher degree of conversion for this monomer [34]. On the other hand, it can be observed that, with respect to the basic composition of the monomer matrix, proportionally more Bis-EMA elutes from the self-curing composite (Table 3). Comparing the structures of Bis-EMA and Bis-GMA, Bis-EMA could show a higher trend to elute due to the lack of hydrogen bonds in the network, as the network is forced apart by the media and thus trapped residual monomers could elute [32, 36]. However, this observation only refers to the self-curing material. The two 3D printing materials, on the other hand, show a proportionally increased elution of Bis-GMA.

In view of the possible cytotoxicity of the monomers a few assumptions have to be stated. The duration of use of temporary crowns and bridges can vary considerably. This can range from a few days to several months. For the following evaluation of the monomer release, the time points 10 days and 6 months are selected, for an elution in ethanol (worst-case scenario) and compared to literature regarding cell experiments in water-based media. A EC_50_ value of 1.83 (1.46–2.30) mmol/L for TEGDMA was stated cytotoxic against human bronchoalveolar carcinoma-derived A549 cells [37]. In the present study the maximum TEGDMA was released from the self-curing composite which reaches concentrations of 0.18 µM after 10 days and 0.89 µM after 6 months. Kraus et al. [38] tested the cytotoxicity of Bis-GMA and TEGDMA against two osteoblast-like cell lines from tumor origin and immortalized human fetal osteoblasts. They found Bis-GMA to be more cytotoxic than TEGDMA. All concentrations used had a cytotoxic effect on the cells, with the lowest concentration to be 0.05 mM. According to this the measured concentration of TEGDMA is below this level just as for Bis-GMA, which reaches concentrations of 0.42 µM after 10 days and 3.48 µM after 6 months. The measured maximum concentration for Bis-EMA reaches concentrations of 0.91 µM after 10 days and 7.17 µM after 6 months. No equivalent data could be found for Bis-EMA in literature. However, the value is at least one power of ten below the values of the other monomers, so a cytotoxic level is probably not reached here either. It should be noted that both the amount of saliva flow and its composition are not considered here as well as the factors influencing these like time of day, food intake and the health status of the test individuals [39–41]. In order to make a definite statement regarding the cytotoxicity of the materials used here, would have to be tested against different cell lines. In addition to the three monomers of interest, other substances have been shown to elute from the samples during storage in ethanol and ethanol/water.

**Fig. 5:**
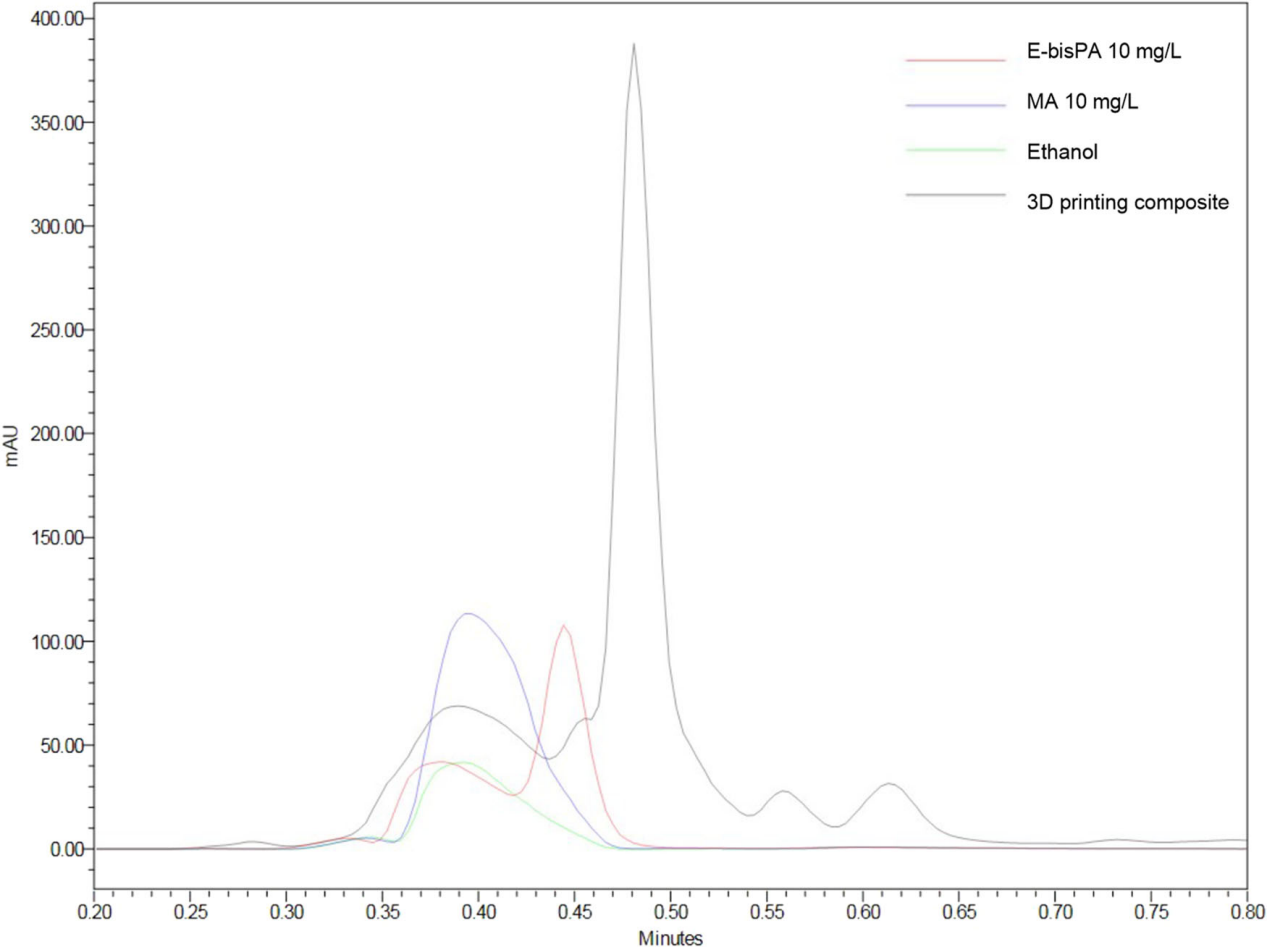
Chromatograms recorded by HPLC-DAD at 205 nm of co-eluting E-bisPA (red), MA (blue) and Ethanol (green) in comparison to an eluate from 3D printing composite (black)

Since the 3D printed samples were washed with isopropanol in post-treatment, there was a possibility that the unknown substance II at retention time 0.49 min could be isopropanol. This has been tested via reference HPLC measurements and can be excluded. These other substances could be additives such as initiators or stabilizers. Elution of possible degradation products such as methacrylic acid (MA), TEGMA (triethylene glycol methacrylate), BisHPPP (bishydroxypropoxy phenyl propane) or E-bisPA (ethoxylated bisphenol-A) also seem plausible [42]. In this context, MA as possible degradation product of all three monomers and E-bisPA as model degradation product of Bis-EMA [42] were tested as co-eluents with the HPLC method used. The resulting chromatogram for the period up to 0.8 min retention time is shown for MA, E-bisPA, pure ethanol and a sample of the 3D printed composite (storage time 59 d) in Fig. 5. The peak at 0.39 min caused by ethanol is enhanced by MA. This indicates a simultaneous elution of the substance with the solvent. E-bisPA generates a peak at 0.45 min, which could be responsible for the shoulder at the peak of the unknown substance of retention time 0.49 min (cf. Fig. 1). This indicates that at least the two substances could be present in the eluates of the composites tested. For a closer look at the unknown peaks, a mass spectroscopic analysis would be beneficial.

The materials currently used on the market vary not only in their manufacturing process but also in the filler content and are thus hardly comparable. In the case of temporary CAD/CAM materials for subtractive manufacturing, materials with different filler contents like 14 wt. % in VITA CAD-temp (Vita) [43] or 27 wt. % in Structur CAD (VOCO) [44] are represented on the market. The self-curing materials also range from lower filled materials to higher filled ones of around 44 wt. %, as in the case of Luxatemp Star (DMG) [43, 45]. Resins for additive manufacturing via SLA and DLP processes are currently found on the market without fillers or the filler content is not given by the manufacturer. The 3D-printing material VarseoSmile Crown (Bego), as a material for permanent crowns, has a filler content of between 30 and 50 wt. % [46]. In the current study the production method can be assessed on the basis of the residual monomer elution. Since all tested materials are built up from the same monomer matrix, a direct comparison can be made between the series of samples. At all measurement times, the self-curing composite exhibited significantly higher monomer elution than all other materials investigated. In industrial processing, higher pressures and temperatures can be used for polymerization (CAD/CAM blank) than for in-situ polymerization (self-curing composite) in the patient’s mouth. Furthermore, during the in-situ processing the material is exposed to oxygen, which can inhibit radical polymerization. In the case of the self-curing material, this increased elution can be attributed to the manufacturing method. In direct comparison, the CAD/CAM blanks hardly showed any quantitatively detectable elution, although the matrix and the initiator system were identical. A higher degree of conversion was also measured for the CAD/CAM blank. This indicates that the CAD/CAM blank achieves a higher degree of crosslinking in industrial processing than the same material when used as a self-curing system. A possible reason for this is the formation of pores in self-curing material observed by Schulz et al. [32], which leads to a heterogeneous material structure with less DC, as also determined via FTIR measurements, here. In contrast, with regard to elution, a homogeneous material structure as with the CAD/CAM blanks should be aimed for [47]. According to Schulz et al. [32], a homogeneously crosslinked polymer matrix reduces elution. In the case of a heterogeneous material composition with the possibility of cluster formations, residual monomer can elute from these more easily. The amounts of eluted monomers from both 3D printing materials were also lower than those of the self-curing material. It appears that this relatively new method of processing can also achieve higher degrees of crosslinking as can be seen for the 3D printing composite, which is with regards to DC comparable to the CAD/CAM blanks. The unfilled material for additive manufacturing had lower DC than those of the CAD/CAM blanks. However, the measured DC were not significantly different from those of the self-curing material. Thus, the reduced elution in comparison to the self-cured materials here cannot be explained by the degree of conversion, as was the case, for example, in the studies by Lempel et al. [48]. This indicates another effect that either accelerates monomer release from the self-curing material or slows it down from the 3D printing resin. The post-treatment of the 3D printed samples (post-curing and washing in isopropanol) could play an important role here. During the washing process in isopropanol non-crosslinked monomers near the surface could have dissolved.

It is possible that a more pronounced correlation between DC and eluting residual monomers would occur if a total extraction were performed instead of a leaching experiment. In order to achieve a complete extraction of all residual monomers, which was not the focus of this study, other extraction methods and solvents would have to be selected. Either the solid samples would have to swell strongly in the solvent so that the sterically hindered uncrosslinked monomers in the network can be released, or the solids would have to be processed before extraction, e.g., by mechanical grinding. In the case of dental composites, however, complete extraction is hardly to be expected. As shown in a previous study on the extraction of antimicrobial agents from restorative composites, even these unbound substances cannot be completely extracted after mechanical grinding in an optimized solvent [49].

With regards to the kinetics of the DC (FTIR serial measurement) the unfilled 3D printing resin cured faster than the 3D printing composite under the here performed conditions. The initiation of the photopolymerization of the 3D printing materials was done during the FTIR serial measurement with a light source, which did not correspond to the one in a DLP printer, likewise, the layer thickness of the applied material did not correspond to the layer thickness in a typical printing process (between 25 and 100 µm; here 50 µm) as also the time of light emission. Due to this the DC measured in the kinetics determinations here cannot be directly compared to the DC of the 3D printed samples. Nevertheless, the kinetics of the DC are comparable for the two materials. For all three measured materials (self-curing composite and the two 3D printing materials) the reaction rate decreases with increasing crosslinking due to the increase of the glass transition temperature. By increasing the reaction temperature, there is the possibility of further curing and thus increasing the DC. In the industrial process (e.g., in the production of the CAD/CAM blanks) these parameters are all optimized to achieve an optimal result. The exact industrial process or the in-situ processing could not be reproduced for any of the materials within the scope of the FTIR series measurement. Furthermore, the two 3D printing materials differ in their filler content (0 and 50 wt.%), resulting in a significantly reduced final DC for the unfilled material. On the one hand, this could possibly be explained by increased quantum yield due to increased light scattering from the filler particles, which does also depend on their geometry [50]. This would lead to altered kinetics in the polymerization. On the other hand, this could be explained by the fact that the monomers form bonds to the surfaces of the fillers. By forming a network, the fillers thus act as a steric hindrance to the elution of residual monomers. As can be observed here, more monomer eluted from the unfilled material than from the composite. If the elution amount in ethanol after 10 d (Table 2) of the two materials is calculated in proportion to the filler content, the unfilled resin also shows a higher monomer elution for all three monomers. For TEGDMA, there is only a slight difference. For the monomers Bis-GMA and Bis-EMA, however, this is much more pronounced. Here, leaching from the unfilled resin is almost twice as high as in the filled composite.

Especially the 3D printing composite evidences promising results in the conducted investigations. It would be interesting to examine whether the processing method of additive manufacturing, including post-processing, could be refined to such an extent that the materials perform as well as or even better than the CAD/CAM blanks in terms of residual monomer elution. The use of 3D printed temporary crowns and bridges made of composite material appears to be superior to the use of a self-curing composite with regard to residual monomer release and DC. However, the use of this class of materials must be verified by other parameters necessary for the application, such as water absorption, strength, hardness or aging.

## Conclusion

Regarding the hypothesis (i) an elution of the matrix monomers from cured samples into the solvents was qualitatively observed and could be quantified for the solvents ethanol and ethanol/water. No quantification was possible for storage in water. Due to this the hypothesis (ii) is only partly given, the eluted monomer concentration depended on the storage medium when ethanol or a mixture of ethanol/water are compared to water. In direct comparison between the media ethanol and ethanol/water such a dependence cannot be seen in general for all monomers and fabrication methods used. The elution of the residual monomers depended on the fabrication method used as stated in hypothesis (iv). The CAD/CAM blanks showed the least monomer release, followed by the 3D printing composite and the 3D printing resin. The self-curing composite showed the most leaching of residual monomers. Regarding the DC, the hypothesis (iii) is only partly given; the composites for milling (CAD/CAM blanks) and 3D printing showed no difference in DC. Although both the monomer release and the DC are dependent on the manufacturing method, there is no direct correlation between the monomer release and the DC. Besides the total amount of residual monomers, further factors as network density and structure seem to influence the monomer elution. This also includes hypothesis (v), in which we stated a dependence of monomer elution and DC on the filler content. This can only be investigated for the 3D printing materials (composite and resin). The 3D printing composite (50% filler content) showed a higher DC and lower monomer release compared to the unfilled resin.

Finally, we can state that manufacturing influences the DC and also the residual monomer release while the latter is not only defined by the DC but other factors as network density and structure. As conclusion, monomer release needs to be characterized individually for each manufacturing method. Likewise, this must be done anew whenever a parameter changes in the manufacturing process or, as in the case of 3D printing, in post-processing.
